# Changes in Sediment Fatty Acid Composition during Passage through the Gut of Deposit Feeding Holothurians: *Holothuria atra* (Jaeger, 1883) and *Holothuria leucospilota* (Brandt, 1835)

**DOI:** 10.1155/2016/4579794

**Published:** 2016-03-02

**Authors:** Prosper L. Mfilinge, Makoto Tsuchiya

**Affiliations:** ^1^Department of Aquatic Sciences and Fisheries Technology, University of Dar es Salaam, P. O. Box 35064, Dar es Salaam, Tanzania; ^2^Laboratory of Ecology and Systematics, Faculty of Science, University of the Ryukyus, Senbaru-1, Nishihara, Okinawa 903-0213, Japan

## Abstract

Sea cucumbers* Holothuria atra* and* Holothuria leucospilota* play an important role in the bioturbation of sediment in coral reef and rocky intertidal ecosystems. This study investigated changes in sediment fatty acid (FA) composition during gut passage in* H. atra* and* H. leucospilota. *The FA composition did not differ significantly between species. Comparison of FA composition in ambient sediment (AS), foregut (FG), midgut (MG), hindgut (HG), and faecal pellets (FPs) indicated that marked changes in FA composition occurred during passage through the gut of* H. atra* and* H. leucospilota.* Saturated fatty acids (SAFAs), monounsaturated fatty acids (MUFAs), polyunsaturated fatty acids (PUFAs), and branched fatty acids (BrFAs) were significantly higher in FG than in AS, suggesting that both species selectively ingested nutrient rich particles. Significant reduction of SAFAs, MUFAs, PUFAs, and BrFAs occurred in MD and HD, with complete elimination of most PUFAs in FPs. A decrease in PUFAs 20:5*ω*3, 18:4*ω*3, 22:5*ω*3, 22:6*ω*3, 18:2*ω*6, 18:3*ω*3, 18:3*ω*6, odd-numbered BrFAs, and MUFA 18:1*ω*7 indicated that algal detritus and bacteria were important part of diet. These results have implications for the fate of specific dietary FAs, especially *ω*3 and *ω*6, and the contribution holothurian FPs make to the FA composition of coral reef and rocky intertidal ecosystems.

## 1. Introduction

The feeding activity of holothurians plays an important role in the bioturbation of sediments [1, 2] by removing substantial amount of organic matter during gut passage [3]. This sediment reworking activity is essential, in order to keep coral reef ecosystems clean and healthy. It has been shown that the efficiency of processing of the sediment and the amount of reworked sediment depends on the length and morphology of the digestive tract, particle sizes, and digestive speed of the ingested sediments [2]. Because these features vary among the holothurians species, sediment reworking efficiency may be species specific.

Holothurians feed on various organic detritus, bacteria, cyanophyceans, and foraminiferans [2], which are rich sources of dietary lipids, particularly FAs. FAs have been used as biomarkers to identify sources and fate of organic matter in marine environments [4–8], due to their structural diversity and high biological specificity [9]. Bacterially derived FAs such as 18:1*ω*7 are common in sediments and are characteristic of organic matter that has been modified and reworked by microbial communities. Polyunsaturated fatty acids (PUFAs) are useful lipid fractions, easily modified by changing conditions, and have stable molecular structures, creating a very distinct signature that allows accurate identification of their various sources [10]. For example, 20:5*ω*3 and 22:6*ω*3 are found in higher quantities in diatoms and dinoflagellates and have been used as diatom and dinoflagellate markers in aquatic environments, respectively [7, 8, 11, 12].

Many studies have investigated composition of FA in tissues of different sea cucumbers, for trophic [13], nutritional [14–16], and medicinal values [16]. None has investigated changes in FA composition when sediment particles transit the gut of holothurians. Information is available for organic carbon [13] and FA composition transiting gut of the copepod,* Calanus helgolandicus* [17]. Understanding of qualitative changes in organic composition of plant detritus as it passes through the gut of sea cucumbers is important in order to identify organic inputs and their fate in marine sediments. This study investigated changes in FA composition during passage through the gut of* Holothuria atra* and* Holothuria leucospilota.* FA composition in the ambient surface sediment (AS) and faecal pellets (FPs) was compared with those in the fore gut (FG), midgut (MG), and hindgut (HG).

## 2. Materials and Methods

### 2.1. Collection of Samples

All samples were collected from Minatogawa, an intertidal rocky reef flat in the southern coast of Okinawa Island, Japan (26°N, 128°E). The study area sediment characteristic is composed of abundant coral fragments > 2 mm mixed with coarse sediment (0.25–2 mm) above a rocky reef substratum. A total of 12 individual species of* Holothuria atra* and* Holothuria leucospilota* were randomly selected while feeding in the rocky pools. Then sediment samples immediately in front of each individual* H. atra* and* H. leucospilota* were taken by scrapping only the top few millimetres. Fresh faecal pellets behind each feeding individual species were gently collected by using a clean spatula. Sampling was done in the late afternoon, when both species were actively feeding. In the laboratory, about 12 g of dry weight of sediment was taken from FG (the first 10% of the anterior intestine), MD (the rest of the intestine), and HG (the last 10% of the posterior intestine) of dissected specimens of* H. atra* and* H. leucospilota.* Six specimens for each species having approximately equal weight: 150–200 g for* H. atra* and 300–400 g for* H. leucospilota,* were used. All samples were stored at −40°C, and lipid analysis was done immediately.

### 2.2. Lipid Extraction

Samples of sediments from the AS, FG, MG, HG, and FPs of each species were extracted in three replicates, following a slightly modified version of the method of Bligh and Dyer [18]. Lipids were extracted by homogenization for 2 min followed by ultrasonication for 20 min with a mixture of distilled water : methanol : chloroform (1 : 2 : 1, 20 cm^3^, v : v : v). The addition of distilled water : chloroform mixture (5 : 5 cm^3^, v : v) formed an aqueous-organic 2-layer system. The lipids migrated into the lower chloroform phase and separation was enhanced by centrifugation (2000 rpm). The extracted lipids were filtered through a glass funnel lined with a precombusted GF/C filter, which removed any fine sediments or particulate matter [19]. The filtrate was concentrated by rotary evaporation and made up to 2 mL.

### 2.3. Saponification and Preparation of FA Methyl Esters (FAMEs)

Lipid extracts were dried under nitrogen and then saponified under reflux (2 h, 100°C) with a 2 mol dm^−3^ NaOH solution in methanol and distilled water (2 : 1, v : v). After acidification with an ultra-pure HCL solution (37.5%), 2 × 2 cm^3^ of chloroform was successively added to recover the lipids. The solvent was then evaporated under a nitrogen stream, and FAs were converted to methyl esters under reflux using 1 mL of 14% BF_3_-methanol for 10 min. Saponification and methylation were done according to Meziane et al. [7] in order to obtain total FAs. FA methyl esters (FAMEs) were purified by high performance thin layer chromatography technique (HPTLC) using Merck plates coated with silica gel (Darmstadt, German). The solvents used for developing were a mixture of hexane/diethyl ether/acetic acid (70 : 30 : 1). Bands containing FAMEs were scraped and collected in a mixture of chloroform/methanol (2 : 1, v : v) at 40°C for 60 min. FAMEs were then isolated in the same solution until analysis by gas chromatography. For all samples, a second plate was prepared in order to estimate the proportion of FAMEs in the total lipids [20]. After drying, the plate was scanned using a flatbed scanner (Epson GT-9000) and Adobe Photoshop software (Adobe systems). The resulting image file was imported into NIH image version 6, to estimate the relative contribution of the FAs, as a proportion of total lipid, by integrating the chromatogram.

### 2.4. Analysis of FAs

The FAMEs were separated and quantified by gas chromatography (GC 14.B, Shimadzu) equipped with a flame ionization detector. Separation was performed with an FFAP-polar capillary column (30 m × 0.32 mm internal diameter, 0.25 *μ*m film thickness) with hydrogen as a carrier gas. After injection at 60°C, the oven temperature was raised to 150°C at a rate of 40°C min^−1^ and then to 230°C at 3°C min^−1^ and finally held constant for 30 min. The flame ionization was held at 240°C. Most FAME peaks were identified by comparing their retention times with those of authentic standards (Supelco Inc., Bellefonte, PA, USA). The standards used were PUFA 1 and PUFA 3 methyl esters; SAFA methyl esters for both even and odd carbon straight chains; palmitic acid; bacterial acid methyl esters; and unsaturated methyl esters. The FAME standards were injected before a sample. For some samples, peaks of FAs were identified with a GC-mass spectrometry. FAs are designated as *X*:*YωZ*, where *X* is the number of carbon atoms, *Y* is the number of double bonds, and *Z* is the position of the ultimate double bond from the terminal methyl group [5].

### 2.5. Statistical Analyses

Similarity and dissimilarity in FA composition between species and at different positions along the gut of* H. atra* and* H. leucospilota* including AS and FPs were tested using one-way analysis of similarity (ANOSIM) with 5,000 permutations using PRIMER software [21]. Data matrices (FA composition in FG, MG, HG, AS, and FPs samples) were used to create triangular similarity matrices based on the Bray-Curtis similarity coefficient, followed by nonmetric multidimensional scaling (n-MDS). Two-way multivariate analysis of variance (MANOVA) (with species,* H. atra* and* H. leucospilota*, and gut positions FG, MG, and HG including AS and FPs entered as fixed effects factors) was used to compare changes in concentration of SAFAs, MUFAs, PUFAs, and BrFAs in the sediment during passage through the gut of* H. atra* and* H. leucospilota.* Any significant species or gut position effects were further examined using Fisher's protected least significant difference (PLSD). All statistical analyses were performed using Stat View 5 software (SAS Institute Inc.). Statistical significance was determined when *p* ≤ 0.05 and data were not transformed.

## 3. Results

### 3.1. FA Composition and Concentration between Species

**Figure 1:**
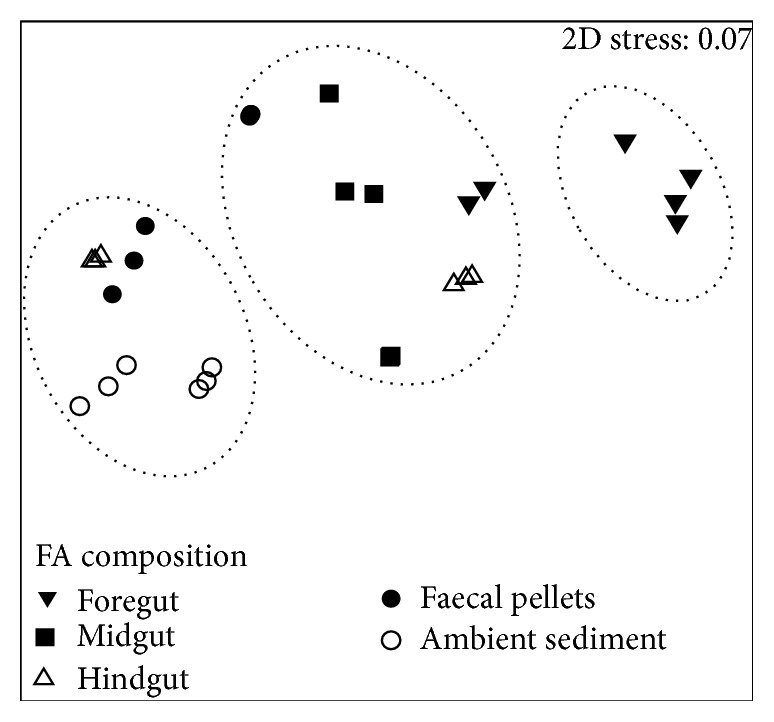
Nonmetric multidimensional scaling plot of the fatty acid composition (*μ*g g^−1^) of ambient sediment, foregut, midgut, hindgut, and faecal pellets of* Holothuria atra* and* Holothuria leucospilota*.

**Figure 2:**
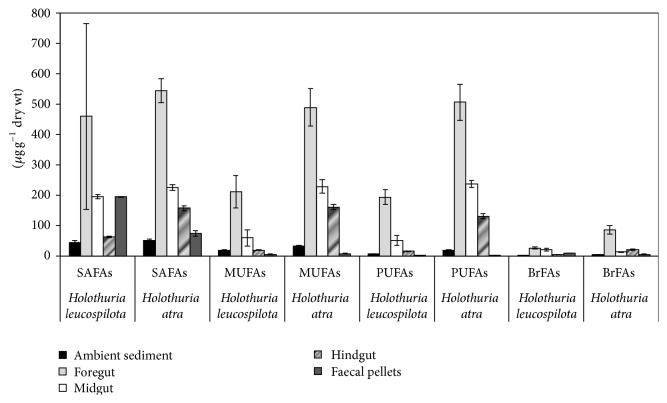
Concentration of SAFAs, MUFAs, PUFAs, and BrFAs in ambient sediment, foregut, midgut, hindgut, and faecal pellets of* Holothuria atra* and* Holothuria leucospilota*. Values are means (± SD).

The results indicated that FA composition and concentration of the ingested sediments changed during passage through the FG, MG, and HG and after release in the FPs for both* Holothuria atra* and* Holothuria leucospilota*. One-way ANOSIM indicated similarity in FA composition between* H. atra* and* H. leucospilota* (Figure 1). But the concentration of SAFA, MUFA, PUFA, and BrFAs differed significantly between species,* H. atra* and* H. leucospilota,* and the gut positions (FG, MG, and HG including AS and FPs) (MANOVA, Pillai's trace, *p* < 0.0001). Significantly higher concentration of MUFA, PUFA, and BrFAs was found in* H. atra* than* H. leucospilota* (ANOVA, *p* < 0.0001), except SAFA (ANOVA, *p* < 0.5856). In addition, MANOVA indicated a significant interaction between species and gut positions (MANOVA, Pillai's trace, *p* < 0.0001), which indicated that both species and position of sediment along the gut affect the concentration of SAFA, MUFA, PUFA, and BrFAs (Figure 2).

### 3.2. FA Composition and Concentration in AS, FG, MG, HG, and FPs

**Figure 3:**
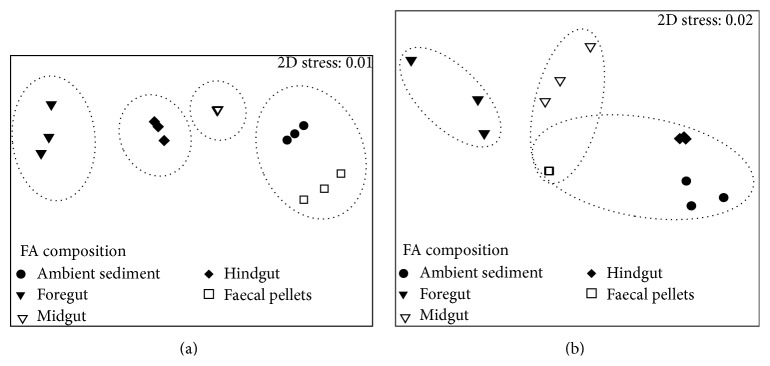
Nonmetric multidimensional scaling plot of the fatty acid composition (*μ*g g^−1^) of ambient sediment, foregut, midgut, hindgut, and faecal pellets of* Holothuria atra* (a) and* Holothuria leucospilota* (b).

The FA concentrations differed significantly among samples: AS, FG, MG, HG, and FPs (ANOVA, *p* < 0.0001) of both species (Figure 2). Analysis of Bray-Curtis similarities followed by n-MDS ordination revealed clear differences in FA composition during passage through the gut of both species of sea cucumbers (Figure 3). One-way analysis of similarity ANOSIM showed a significant difference in FA composition between the AS and gut positions for* H. atra *(*R* = 0.995, *p* < 0.001) and (*R* = 0.975, *p* < 0.001) for* H. leucospilota*. In particular, there was a distinct dissimilarity in FA composition among samples of AS with FG, FG with HG, and HG with FPs of* H. atra *(Figure 3(a)) and among samples of AS with FG and FG with HG of* H. leucospilota *(Figure 3(b)). However, there was no significant difference in FA composition between FPs and AS for* H. atra *(Figure 3(a)) and between AS and HG, AS and FPs, FPs and HG, and FPs and MD for* H. leucospilota* (Figure 3(b)).

Along the gut, the concentration of SAFAs, MUFAs, PUFAs, and BrFAs differed significantly between the AS and FG of both* H. atra* and* H. leucospilota* (Fisher's PLSD, *p* < 0.0001). This was followed by a significant reduction of all the FA groups (SAFAs, MUFAs, PUFAs, and BrFAs) which occurred in the MD, HG, and FPs, except for an increase in SAFAs in the FPs of* H. leucospilota *(Figure 2). SAFAs were mostly contributed by 14:0, 16:0, and 18:0 and least contributed by long chain SAFAs of >24 carbon atoms (Tables 1 and 2). The concentration of 16:0 was highest in the FG of both species and lowest in the FPs for* H. atra* and in the HG for* H. leucospilota.* MUFAs were mainly contributed by 16:1*ω*9, 16:1*ω*7, 18:1*ω*9, and 18:1*ω*7, which increased significantly in the FG, and were reduced significantly in the HG and FPs of both species. MUFAs 18:1*ω*9 and 18:1*ω*7 were completely eliminated in the FPs of* H. leucospilota. *The concentration of BrFAs was mostly contributed by 15:0 iso and 17:0 anteiso. While 15:0 iso was released unchanged, 17:0 anteiso was completely eliminated in FPs.

PUFAs were dominated by 18:2*ω*6, 18:3*ω*3, 18:3*ω*6, 18:4*ω*3, 20:4*ω*6, 20:5*ω*3, and 22:6*ω*3. Their concentration was significantly higher in FG than in AS. PUFAs showed a significant decrease in the MG and HG. At the end, 18:2*ω*6, 18:3*ω*3, 18:4*ω*3, 20:4*ω*6, and 22:6*ω*3 were not detected in the FPs of* H. atra* (Table 1). The PUFAs 18:2*ω*6, 18:3*ω*6, 20:4*ω*6, 20:5*ω*3, and 22:6*ω*3 were not detected in the HG and FPs of* H. leucospilota *(Table 2). The *ω*3 and *ω*6 PUFAs detected in the samples comprised the essential FAs 18:2*ω*6 (Linoleic acid) and 18:3*ω*3 (*α*-Linolenic acid); others were 18:3*ω*6, 18:4*ω*3, 20:4*ω*6, 20:3*ω*3, and 20:4*ω*6 Arachidonic acid (AA), 20:5*ω*3 eicosapentaenoic acid (EPA), 22:4*ω*3, 22:5*ω*6, and 22:5*ω*3 docosapentaenoic acid (DPA), and 22:6*ω*3 docosahexaenoic acid (DHA). These FAs were detected in low concentration in the AS of both species. However, like other FAs, concentration increased in the FG of both species, declined significantly in the HG, and was completely eliminated in the FP (Tables 1 and 2).

## 4. Discussion

### 4.1. Comparison of FA Composition of* Holothuria atra* and* Holothuria leucospilota*

The aim of this study was to investigate changes in FA composition during passage through the gut of* H. atra *and* H. leucospilota.* Results indicate that marked changes in sediment FA composition and concentration occurred during passage of the sediment particles through the gut of both holothurian species and that no difference in gut sediment FA composition was found between the two species. Studies show that the major source of lipids in marine sediments is organic detritus [9, 22], while feeding holothurians ingest various organic detritus, such as bacteria [23, 24], cyanophyceans, phytodetritus, and foraminiferans [2, 13], which are rich sources of dietary lipids, especially FAs [8, 13, 25]. The similarity in the gut sediment FA composition found in* H. atra *and* H. leucospilota* was mainly due to ingestion of the same type of organic material [26] (Figure 1). This was revealed by detection of FA biomarkers for bacteria 15:0 and 17:0 iso and anteiso, 16:1*ω*7 and 18:1*ω*7 [22], diatoms 20:5*ω*3 [27], dinoflagellates 18:4*ω*3, 22:5*ω*3, and 22:6*ω*3 [28, 29], and green macroalgae 18:2*ω*6, 18:3*ω*3, and 18:3*ω*6 [30] in the FG of* H. atra* and* H. leucospilota*. The only difference in FAs between* H. atra* and* H. leucospilota* occurred in the concentration of MUFA, PUFA, and BrFAs (Figure 2). Significantly higher concentration of MUFA, PUFA, and BrFAs was found in* H. atra* than* H. leucospilota,* possibly attributed to ingestion of organic rich particles, because they contain higher levels of lipids and FAs [8]. Preference to organic rich particles by* H. atra* compared to* H. leucospilota* was also shown by Mangion et al. [26], where sediments along the gut of* H. atra* were found to contain higher levels of organic carbon than* H. leucospilota*, indicating that* H. atra* selectively ingested particles which were rich in organic matter.

### 4.2. Changes in FA Composition and Concentration between AS and FG

The FA composition of AS differed significantly from the FA composition of FG in both* H. atra *and* H. leucospilota* (Figure 3). This difference was also indicated by higher concentration of SAFAs, MUFAs, PUFAs, and BrFAs in FG than in AS, suggesting specific selection of organic rich particles commonly exhibited by holothurians during feeding [31, 32] (Figure 2). SAFAs were mostly contributed by palmitic acid 16:0, which occurs ubiquitous in nature [5]. High concentration of 16:0 in sediments is an indication of fresh detritus [8, 25]. In a field experiment, Mangion et al. [26] found that C : N ratio of sediment ingested by* H. atra* and* H. leucospilota* decreased between the adjacent sediment and FG, as a result of ingestion of particles with fresh organic matter. In this study highest concentration of 16:0 was found in FG, an indication that the holothurians consumed fresh organic matter (Tables 1 and 2). The decrease in 16:0 in MD and HG indicated that substantial organic matter degradation occurred during gut passage. However, the low detection of even long chain SAFAs > 24 carbon atoms (26:0, 28:0, and 32:0) biomarkers of vascular plants [8] in AS and their complete absence in MG, HG, and FPs showed that vascular plant detritus was not an important part of the diet of* H. atra* and* H*.* leucospilota*.

High concentrations of MUFAs and odd BrFAs suggest an increase in bacteria [33]. This was indicated by higher concentration of bacterial markers, such as 15:0 and 17:0 iso and anteiso and MUFAs 16:1*ω*7 and 18:1*ω*7 in FG than in AS (Tables 1 and 2), an indication of higher bacteria content in the FG than AS. Similar findings were made by Moriarty [23], suggesting that* H. atra* and* H*.* leucospilota* preferentially selected and ingested bacterial rich particles. This study also found higher PUFA concentration in FG than in AS. Elevated levels of PUFAs in sediments indicate presence of undegraded organic phytodetritus [34, 35], because these plants contain large proportions of PUFAs (Linoleic acid 18:2*ω*6, *α*-Linolenic acid 18:3*ω*3 and 18:3*ω*6, eicosapentaenoic acid 20:5*ω*3, and docosahexaenoic acid 22:6*ω*3) in their fresh state [5, 8, 27, 30, 36]. For example, a study by Uthicke [31] found more dead diatoms inside* H. atra *and* S. chloronotus* gut than in adjacent sediment. Therefore, the change in FA composition and concentration between AS and FG was attributed by increase in FA biomarkers for bacteria, diatoms, dinoflagellates, and green macroalgae.

Results further suggest specific selection of algal [13] and bacteria rich detrital particles [13, 37].

### 4.3. Changes in FA Composition and Concentration in FG, MD, HG, and FPs

The FA composition of FG differed significantly from that of MG, HG, and FPs for both species (Figure 3). This was accompanied by a significant decrease in SAFAs, MUFAs, PUFAs, and BrFAs in MG, HG, and FPs. FPs had the lowest concentration of PUFAs, MUFAs, and BrFAs (Figure 2). However, the similarity in FA composition between FPs and AS and the substantial decrease in concentration of most FAs indicate absorption of some of the organic derived FAs in the tissue during gut passage. Since decline in 16:0 indicates loss of organic matter [25], low concentrations of 16:0 in HG and FPs possibly show that significant processing of organic matter occurred in the gut of* H. atra *and* H. leucospilota*. The significant decrease of the diatom biomarker 20:5*ω*3, the specific dinoflagellate biomarkers 18:4*ω*3, 22:5*ω*3, and 22:6*ω*3, and the green macroalgae biomarkers 18:2*ω*6, 18:3*ω*3, and 18:3*ω*6 in the FA composition of MD and HG (Tables 1 and 2) was of particular interest. Large proportions of 22:6*ω*3 and 20:5*ω*3 have been detected in tissues of* H. leucospilota* [15] and* Apostichopus japonicus* [14] and in abyssal holothurians [13, 38]. Thus the significant decrease of the algal biomarkers along the gut of* H. atra *and* H. leucospilota* indicates a greater algal input to the diet and assimilation into tissues. For example, assimilation efficiency of 46.9% for diatoms of a holothurian species from California was reported by Yingst [39]. The complete elimination of algal biomarkers in FPs of* H. atra *and* H. leucospilota* further suggests that the holothurians selectively absorbed PUFA compounds during digestion.

The decrease in PUFA concentration corresponded with low levels of MUFA and BrFAs. In particular, there was a significant decrease in BrFAs 15:0 and 17:0 iso and anteiso and MUFAs 16:1*ω*7 and 18:1*ω*7 markers of bacteria, during gut passage. Lowest concentrations were detected in HG and FPs (Tables 1 and 2). Since holothurians ingest and digest a variety of bacteria [2, 37], detection of bacterial derived FAs in the gut indicates a substantial bacterial input to the diet [40]. FAs biomarkers of bacteria have also been detected in holothurian tissue samples [13]. Low concentration of the biomarkers in HG and FP indicate bacterial assimilation into tissues of* H. atra* and* H. Leucospilota* and absorption of bacterial FA compounds during digestion.

The FA composition of FP was similar to the FA composition of AS for* H. atra*, while for* H. leucospilota* the FA composition of FP was different from that of AS (Figure 3). The similarities and differences in FA composition revealed by n-MDS suggest species specific differences in the digestion and absorption of organic matter [2, 26]. The near depletion of some of the dietary FAs in the FP indicates that holothurians, like other invertebrates, selectively absorb FAs, especially PUFAs for their metabolic activities [40].

## 5. Conclusion

FA composition and concentration changed significantly during passage through the gut of* Holothuria atra *and* Holothuria leucospilota*, first because both species specifically selected algal and bacterial rich detrital particles which are also rich in dietary FAs and secondly due to assimilation of the dietary FAs in the tissues. Specific selection to organic rich particles was evidenced by changes in FA composition and increase in concentration of SAFA, MUFA, PUFA, and BrFAs in the FG compared to AS. This increase was also indicated by high levels of FA biomarkers for bacteria, diatoms, dinoflagellate, and green macroalgae, an indication that* H. atra *and* H. leucospilota* selected algal and detrital particles coated with bacteria. The significant decrease of most of the FAs in the HG and the complete elimination of some of the FAs in FPs were possibly due to selective absorption of the FAs during digestion. These findings have implications for nutrition, metabolic functions of holothurians, and the fate of dietary FAs in marine sediments.

## Figures and Tables

**Table 1:** Fatty acid composition (*µ*g g^−1^ dry weight) of ambient sediment, foregut, midgut, hindgut, and faecal pellets of *Holothuria atra*.

Fatty acids	Ambient sediment	Foregut	Midgut	Hindgut	Faecal pellets
14:0	3.7 ± 0.2	41.3 ± 10.4	5.0 ± 0.0	8.9 ± 0.4	5.4 ± 0.7
15:0 iso	0.5 ± 0.0	17.2 ± 2.7	2.3 ± 0.0	1.7 ± 0.1	0.5 ± 0.1
15:0 anteiso	0.2 ± 0.0	2.4 ± 2.4	**—**	**—**	0.2 ± 0.0
15:0	0.6 ± 0.0	10.8 ± 2.0	1.5 ± 0.0	2.0 ± 0.1	0.8 ± 0.1
16:0 iso	0.0 ± 0.0	9.4 ± 1.4	1.4 ± 0.0	0.5 ± 0.4	1.5 ± 0.2
16:0	41.4 ± 2.7	301.8 ± 22.1	112.8 ± 9.8	109.6 ± 5.0	54.4 ± 6.7
16:1*ω*9	20.9 ± 1.4	174.0 ± 11.7	130.8 ± 0.3	65.3 ± 3.0	1.7 ± 0.2
16:1*ω*7	1.0 ± 0.1	12.4 ± 0.7	12.5 ± 0.0	2.1 ± 0.1	1.0 ± 0.1
17:0 iso	0.8 ± 0.0	8.2 ± 8.2	1.1 ± 0.8	1.6 ± 0.1	0.3 ± 0.0
17:0 anteiso	1.7 ± 0.1	21.5 ± 3.0	3.8 ± 0.0	6.6 ± 0.3	**—**
17:0	0.4 ± 0.0	4.7 ± 4.7	1.6 ± 0.0	1.7 ± 0.1	1.0 ± 0.1
17:1	1.1 ± 0.1	45.8 ± 27.5	31.5 ± 20.9	4.6 ± 0.6	1.8 ± 0.2
18:0 iso	0.5 ± 0.0	**—**	**—**	**—**	0.8 ± 0.1
18:0 anteiso	0.3 ± 0.0	27.2 ± 27.2	3.5 ± 0.0	9.8 ± 0.4	0.3 ± 0.0
18:0	4.0 ± 0.3	114.6 ± 7.4	91.3 ± 0.1	19.8 ± 0.9	10.1 ± 1.3
18:1*ω*9	4.9 ± 0.3	44.4 ± 5.1	8.6 ± 0.1	24.4 ± 1.1	0.5 ± 0.1
18:1*ω*7	4.1 ± 0.3	83.2 ± 4.7	15.1 ± 0.1	19.2 ± 0.9	0.4 ± 0.0
18:2*ω*6	1.7 ± 0.1	48.8 ± 4.1	28.3 ± 10.0	16.0 ± 0.7	**—**
18:3*ω*6	0.8 ± 0.1	4.1 ± 4.1	1.0 ± 0.0	0.7 ± 0.6	0.2 ± 0.0
18:3*ω*4	0.0 ± 0.0	5.3 ± 5.3	**—**	2.9 ± 0.1	**—**
18:3*ω*3	1.4 ± 0.1	52.9 ± 2.9	18.1 ± 9.2	11.3 ± 0.5	**—**
18:4*ω*3	1.6 ± 0.1	20.7 ± 4.1	2.1 ± 0.0	1.8 ± 0.1	**—**
20:0	0.3 ± 0.0	19.9 ± 0.9	5.4 ± 0.0	2.9 ± 1.1	0.7 ± 0.1
20:1*ω*9	0.4 ± 0.0	85.3 ± 2.5	17.2 ± 0.1	31.8 ± 1.4	—
20:1*ω*7	0.4 ± 0.0	6.5 ± 6.5	3.0 ± 0.0	3.3 ± 0.1	—
20:2	0.0 ± 0.0	24.5 ± 1.5	5.2 ± 0.0	5.8 ± 0.3	0.1 ± 0.0
21:0	0.2 ± 0.0	—	—	0.4 ± 0.4	—
20:3*ω*6	**—**	**—**	1.3 ± 0.0	**—**	0.1 ± 0.0
20:4*ω*6	2.9 ± 0.2	140.1 ± 5.4	91.6 ± 5.8	52.0 ± 2.4	**—**
20:3*ω*3	0.0 ± 0.0	4.6 ± 4.6	3.0 ± 0.0	1.7 ± 1.5	**—**
20:5*ω*3	7.2 ± 0.5	151.9 ± 1.4	67.6 ± 5.8	30.4 ± 1.4	0.1 ± 0.0
22:0	0.2 ± 0.0	34.5 ± 3.4	5.3 ± 0.0	8.3 ± 0.4	0.7 ± 0.1
22:1*ω*9	0.0 ± 0.0	8.9 ± 8.9	1.2 ± 0.5	4.6 ± 0.2	—
22:1*ω*7	—	—	0.4 ± 0.4	—	—
23:0	0.0 ± 0.0	9.6 ± 4.1	1.3 ± 0.0	2.0 ± 0.1	—
22:4*ω*3	0.1 ± 0.0	**—**	**—**	**—**	**—**
22:5*ω*6	0.2 ± 0.0	5.4 ± 5.4	5.5 ± 0.5	3.8 ± 0.2	**—**
22:5*ω*3	0.2 ± 0.0	2.2 ± 2.2	0.3 ± 0.3	**—**	**—**
24:0	0.4 ± 0.0	4.0 ± 4.0	—	0.7 ± 0.6	1.1 ± 0.1
22:6*ω*3	1.7 ± 0.1	45.3 ± 32.5	12.6 ± 0.0	2.9 ± 0.5	**—**
24:1	0.0 ± 0.0	28.1 ± 23.1	7.9 ± 0.2	5.4 ± 0.2	—
25:0	0.0 ± 0.0	2.3 ± 2.3	1.0 ± 0.0	0.4 ± 0.8	0.5 ± 0.1
26:0	0.1 ± 0.0	—	—	—	—

Values are means ± SD; *n* = 3; — means not detected or traces.

**Table 2:** Fatty acid composition (*µ*g g^−1^ dry weight) of ambient sediment, foregut, midgut, hindgut, and faecal pellets of *Holothuria leucospilota*.

Fatty acids	Ambient sediment	Foregut	Midgut	Hindgut	Faecal pellets
14:0	4.2 ± 0.6	29.7 ± 3.1	7.4 ± 2.0	4.7 ± 0.2	18.6 ± 11.0
14:1	—	0.6 ± 0.6	2.0 ± 2.0	—	—
15:0 iso	0.5 ± 0.1	5.6 ± 3.8	2.3 ± 1.2	0.7 ± 0.0	0.9 ± 0.5
15:0 anteiso	0.7 ± 0.1	3.3 ± 2.6	1.8 ± 1.0	0.7 ± 0.0	0.2 ± 0.1
15:0	0.5 ± 0.1	1.9 ± 1.7	2.8 ± 0.6	1.0 ± 0.0	1.7 ± 0.9
15:1	—	—	4.7 ± 1.9	—	2.1 ± 1.2
16:0 iso	—	4.2 ± 4.3	3.6 ± 3.6	1.4 ± 0.1	3.2 ± 1.8
16:0 anteiso	—	0.6 ± 0.5	—	0.2 ± 0.0	1.4 ± 0.8
16:0	34.8 ± 5.3	309.2 ± 178.0	94.4 ± 7.9	40.3 ± 1.7	151.6 ± 86.7
16:1*ω*9	6.3 ± 1.0	85.0 ± 30.9	27.7 ± 25.8	0.4 ± 0.0	1.4 ± 0.6
16:1*ω*7	0.3 ± 0.6	32.3 ± 29.8	11.9 ± 10.3	16.1 ± 0.7	0.4 ± 0.1
17:0 iso	0.1 ± 0.2	3.5 ± 0.4	7.8 ± 0.4	0.8 ± 0.0	0.6 ± 0.3
17:0 anteiso	0.0 ± 0.0	6.7 ± 6.2	2.8 ± 0.4	—	0.5 ± 0.3
17:0	0.3 ± 0.6	6.7 ± 6.4	7.8 ± 1.3	2.1 ± 0.1	1.3 ± 0.7
17:1	0.6 ± 1.0	1.7 ± 1.6	—	—	1.0 ± 0.6
18:0 iso	0.3 ± 0.5	0.2 ± 0.2	1.8 ± 1.0	—	1.5 ± 0.9
18:0 anteiso	0.1 ± 0.2	1.5 ± 1.4	—	—	0.3 ± 0.2
18:0	5.4 ± 4.0	69.4 ± 77.6	54.2 ± 9.3	8.9 ± 0.4	13.2 ± 7.4
18:1*ω*9	5.1 ± 4.1	32.6 ± 3.9	3.5 ± 1.8	1.0 ± 0.0	—
18:1*ω*7	1.7 ± 1.2	30.7 ± 21.0	1.4 ± 0.3	1.2 ± 0.0	—
18:2*ω*6	0.0 ± 0.0	11.7 ± 1.0	—	—	0.0 ± 0.0
18:3*ω*6	0.1 ± 0.1	9.5 ± 9.2	14.3 ± 3.0	—	—
18:3*ω*4	1.2 ± 1.1	11.1 ± 3.9	2.7 ± 0.9	7.9 ± 0.3	—
18:3*ω*3	0.5 ± 0.8	8.5 ± 1.5	1.8 ± 1.8	0.5 ± 0.0	—
18:4*ω*3	0.5 ± 0.9	36.8 ± 1.4	9.8 ± 9.8	2.5 ± 0.1	—
20:0	0.2 ± 0.4	19.8 ± 19.7	10.2 ± 5.7	2.8 ± 0.1	1.1 ± 0.5
20:1*ω*9	0.0 ± 0.0	15.6 ± 15.7	4.9 ± 4.9	—	—
20:1*ω*7	0.0 ± 0.0	3.0 ± 3.8	—	—	—
20:2	2.5 ± 2.0	3.8 ± 3.6	0.0 ± 0.0	—	—
21:0	0.0 ± 0.0	0.4 ± 0.4	6.9 ± 6.9	—	—
20:3*ω*6	0.0 ± 0.1	16.4 ± 11.8	5.6 ± 4.6	3.7 ± 0.2	—
20:4*ω*6	1.0 ± 1.7	24.1 ± 12.4	—	—	—
20:4*ω*3	0.0 ± 0.1	0.8 ± 0.7	—	—	—
20:5*ω*3	0.2 ± 0.4	44.1 ± 12.2	8.4 ± 8.4	—	—
22:0	0.2 ± 0.2	16.0 ± 17.8	6.6 ± 6.6	2.4 ± 0.1	1.3 ± 0.6
22:1*ω*9	0.0 ± 0.0	0.7 ± 0.6	0.4 ± 0.4	—	—
22:1*ω*7	0.1 ± 0.1	3.9 ± 4.1	0.7 ± 0.7	—	—
22:2	0.0 ± 0.0	0.2 ± 0.2	1.7 ± 1.7	—	0.3 ± 0.2
23:0	0.0 ± 0.0	2.4 ± 3.2	1.3 ± 1.3	—	—
22:4*ω*3	0.1 ± 0.1	6.3 ± 1.3	1.3 ± 1.3	—	—
22:5*ω*6	0.1 ± 0.1	—	1.3 ± 1.3	—	—
22:5*ω*3	0.1 ± 0.2	5.0 ± 1.5	1.1 ± 1.1	—	—
24:0	0.6 ± 1.0	3.1 ± 2.3	1.2 ± 1.2	0.2 ± 0.0	2.3 ± 1.3
22:6*ω*3	0.2 ± 0.0	15.1 ± 3.0	1.7 ± 1.7	—	—
24:1	0.2 ± 0.3	4.3 ± 5.5	1.7 ± 0.6	—	—
25:0	0.0 ± 0.1	—	0.4 ± 0.4	0.3 ± 0.0	2.5 ± 1.5
32:0	0.0 ± 0.0	—	1.5 ± 1.5	—	—

Values are means ± SD; *n* = 3; — means not detected or traces.

## References

[1] Hammond L. S. (1982). Patterns of feeding and activity in deposit-feeding holothurians and echinoids (Echinodermata) from a shallow back-reef lagoon, Discovery Bay, Jamaica. *Bulletin of Marine Science*.

[2] Wiedemeyer W. L., Richmond R. H. Feeding behaviour of two tropical holothurians, *Holothuria* (*Metriatyla*) *scabra* and *H.* (*Halodeima*) *atra* from Okinawa, Japan.

[3] Dar M. A., Ahmad H. O. (2006). The feeding selectivity and ecological role of shallow water holothurians in the Red Sea. *SPC Beche-de-mer Information Bulletin*.

[4] Harvey H. R. (1994). Fatty acids and sterols as source markers of organic matter in sediments of the North Carolina continental slope. *Deep-Sea Research Part II*.

[5] Meziane T., Tsuchiya M. (2000). Fatty acids as tracers of organic matter in the sediment and food web of a mangrove/intertidal flat ecosystem, Okinawa, Japan. *Marine Ecology Progress Series*.

[6] Meziane T., Tsuchiya M. (2002). Organic matter in a subtropical mangrove-estuary subjected to wastewater discharge: origin and utilisation by two macrozoobenthic species. *Journal of Sea Research*.

[7] Meziane T., Sanabe M. C., Tsuchiya M. (2002). Role of fiddler crabs of a subtropical intertidal flat on the fate of sedimentary fatty acids. *Journal of Experimental Marine Biology and Ecology*.

[8] Mfilinge P. L., Meziane T., Bachok Z., Tsuchiya M. (2005). Litter dynamics and particulate organic matter outwelling from a subtropical mangrove in Okinawa Island, South Japan. *Estuarine, Coastal and Shelf Science*.

[9] Parkes R. J. Analysis of microbial communities within sediments using biomarkers.

[10] Sargent J. R., Bell M. V., Hendersen R. J., Tocher D. R., Mellinger J. (1990). Polyunasaturated fatty acids in marine and terrestrial foodwebs. *Animal Nutrition and Transport Processes. 1: Nutrition in Wild and Domestic Animals*.

[11] Hudson I. R., Wigham B. D., Billett D. S. M., Tyler P. A. (2003). Seasonality and selectivity in the feeding ecology and reproductive biology of deep-sea bathyal holothurians. *Progress in Oceanography*.

[12] Wai T.-C., Ng J. S. S., Leung K. M. Y., Dudgeon D., Williams G. A. (2008). The source and fate of organic matter and the significance of detrital pathways in a tropical coastal ecosystem. *Limnology and Oceanography*.

[13] Drazen J. C., Phleger C. F., Guest M. A., Nichols P. D. (2008). Lipid, sterols and fatty acid composition of abyssal holothurians and ophiuroids from the North-East Pacific Ocean: food web implications. *Comparative Biochemistry and Physiology. Part B*.

[14] Yu H., Gao Q., Dong S., Wen B. (2015). Changes in fatty acid profiles of sea cucumber *Apostichopus japonicus* (Selenka) induced by terrestrial plants in diets. *Aquaculture*.

[15] Yahyavi M., Afkhami M., Mokhleci A. (2012). Fatty acid in local sea cucumber species from Persian Gulf (Qeshm Island). *Annals of Biological Research*.

[16] Ridzwan B. H., Hanita M. H., Nurzafirah M., Siti Norshuhadaa M. P., Farah Hanis Z. (2014). Free fatty acids composition in lipid extracts of several sea cucumbers species from Malaysia. *International Journal of Bioscience, Biochemistry and Bioinformatics*.

[17] Prahl F. G., Eglinton G., Corner E. D. S., O'Hara S. C. M., Forsberg T. E. V. (1984). Changes in plant lipids during passage through the gut of *Calanus*. *Journal of the Marine Biological Association of the United Kingdom*.

[18] Bligh E. G., Dyer W. J. (1959). A rapid method of total lipid extraction and purification. *Canadian Journal of Biochemistry and Physiology*.

[19] Wilson S., Burns K., Codi S. (2001). Identifying sources of organic matter in sediments from a detritivorous coral reef fish territory. *Organic Geochemistry*.

[20] Yamashiro H., Oku H., Higa H., Chinen I., Sakai K. (1999). Composition of lipids, fatty acids and sterols in Okinawan corals. *Comparative Biochemistry and Physiology. Part B*.

[21] Clarke K. R., Warwick R. M. (2001). *Change in Marine Communities: An Approach to Statistical Analysis and Interpretation*.

[22] Volkman J. K., Johns R. B., Gillan F. T., Perry G. J., Bavor H. J. (1980). Microbial lipids of an intertidal sediment—I. Fatty acids and hydrocarbons. *Geochimica et Cosmochimica Acta*.

[23] Moriarty D. J. W. (1982). Feeding of *Holothuria atra* and *Stichopus chloronotus* on bacteria, organic carbon and organic nitrogen in sediments of the Great Barrier Reef. *Australian Journal of Marine and Freshwater Research*.

[24] Hammond S. L. (1983). Nutrition of deposit-feeding holothuroids and echinoids (Echinodermata) from a shallow reef lagoon, Discovery Bay, Jamaica. *Marine Ecology Progress Series*.

[25] Mfilinge P. L., Meziane T., Bachok Z., Tsuchiya M. (2003). Fatty acids in decomposing mangrove leaves: microbial activity, decay and nutritional quality. *Marine Ecology Progress Series*.

[26] Mangion P., Taddei D., Conand C., Frouin P., Nebelsick J. H., Heinzeller T. (2004). Feeding rate and impact of sediment reworking by two deposit feeders *Holothuria leucospilota* and *Holothuria atra* on a fringing reef (Reunion Island, Indian Ocean). *Echinoderms: Munchen*.

[27] Pond D. W., Bell M. V., Harris R. P., Sargent J. R. (1998). Microplanktonic polyunsaturated fatty acid markers: a mesocosm trial. *Estuarine, Coastal and Shelf Science A*.

[28] Graeve M., Kattner G., Hagen W. (1994). Diet-induced changes in the fatty acid composition of Arctic herbivorous copepods: experimental evidence of trophic markers. *Journal of Experimental Marine Biology and Ecology*.

[29] Zhukova N. V., Aizdaicher N. A. (1995). Fatty acid composition of 15 species of marine microalgae. *Phytochemistry*.

[30] Napolitano G. E., Pollero R. J., Gayoso A. M., MacDonald B. A., Thompson R. J. (1997). Fatty acids as trophic markers of phytoplankton blooms in the Bahia Blanca estuary (Buenos Aires, Argentina) and in Trinity Bay (Newfoundland, Canada). *Biochemical Systematics and Ecology*.

[31] Uthicke S. (1999). Sediment bioturbation and impact of feeding activity of *Holothuria* (*Halodeima*) *atra* and *Stichopus chloronotus*, two sediment feeding holothurians, at Lizard Island, great barrier reef. *Bulletin of Marine Science*.

[32] Roberts D., Gebruk A. V., Levin V., Manship B. A. D. (2000). Feeding and digestive strategies in deposit-feeding holothurians. *Oceanography and Marine Biology: An Annual Review*.

[33] Bachok Z., Mfilinge P., Tsuchiya M. (2006). Characterization of fatty acid composition in healthy and bleached corals from Okinawa, Japan. *Coral Reefs*.

[34] Carrie R. H., Mitchell L., Black K. D. (1998). Fatty acids in surface sediment at the Hebridean shelf edge, west of Scotland. *Organic Geochemistry*.

[35] Fileman T. W., Pond D. W., Barlow R. G., Mantoura R. F. C. (1998). Vertical profiles of pigments, fatty acids and amino acids: evidence for undegraded diatomaceous material sedimenting to the deep ocean in the Bellingshausen Sea, Antarctica. *Deep Sea Research Part I: Oceanographic Research Papers*.

[36] Johns R. B., Nichols P. D., Perry G. J. (1979). Fatty acid composition of ten marine algae from Australian waters. *Phytochemistry*.

[37] Gao F., Li F., Tan J., Yan J., Sun H. (2014). Bacterial community composition in the gut content and ambient sediment of sea cucumber *Apostichopus japonicus* revealed by 16S rRNA gene pyrosequencing. *PLoS ONE*.

[38] Ginger M. L., Santos V. L. C. S., Wolff G. A. (2000). A preliminary investigation of the lipids of abyssal holothurians from the north-east Atlantic Ocean. *Journal of the Marine Biological Association of the United Kingdom*.

[39] Yingst J. Y. (1976). The utilization of organic matter in shallow marine sediments by an epibenthic deposit-feeding holothurian. *Journal of Experimental Marine Biology and Ecology*.

[40] Parrish C. C. (2013). Lipids in marine ecosystems. *ISRN Oceanography*.

